# *Salmonella* Virulence Effector SopE and Host GEF ARNO Cooperate to Recruit and Activate WAVE to Trigger Bacterial Invasion

**DOI:** 10.1016/j.chom.2012.01.006

**Published:** 2012-02-16

**Authors:** Daniel Humphreys, Anthony Davidson, Peter J. Hume, Vassilis Koronakis

**Affiliations:** 1 Department of Pathology, Cambridge University, Tennis Court Road, Cambridge, CB21QP, UK

## Abstract

*Salmonella* virulence effectors elicit host cell membrane ruffling to facilitate pathogen invasion. The WAVE regulatory complex (WRC) governs the underlying membrane-localized actin polymerization, but how *Salmonella* manipulates WRC is unknown. We show that Rho GTPase activation by the *Salmonella g*uanine nucleotide *e*xchange *f*actor (GEF) SopE efficiently triggered WRC recruitment but not its activation, which required host Arf GTPase activity. Invading *Salmonella* recruited and activated Arf1 to facilitate ruffling and uptake. Arf3 and Arf6 could also enhance invasion. RNAi screening of host Arf-family GEFs revealed a key role for ARNO in pathogen invasion and generation of pathogen-containing macropinosomes enriched in Arf1 and WRC. *Salmonella* recruited ARNO via Arf6 and the phosphoinositide phosphatase effector SopB-induced PIP3 generation. ARNO in turn triggered WRC recruitment and activation, which was dramatically enhanced when SopE and ARNO cooperated. Thus, we uncover a mechanism by which pathogen and host GEFs synergize to regulate WRC and trigger *Salmonella* invasion.

## Highlights

► *Salmonella* GEF SopE recruits the WAVE regulatory complex (WRC) to the membrane ► SopE activation of Rac1 is insufficient for WRC activation which requires Arf GTPase ► *Salmonella* recruits the host Arf GEF ARNO via Arf6 and effector SopB-induced PIP3 ► *Salmonella* SopE and ARNO cooperate to recruit and activate WRC and trigger invasion

## Introduction

Rho GTPases are anchored to membranes where they initiate membrane-associated actin filament polymerization to drive key cell processes, including motility, membrane ruffling, and macropinocytosis (Ridley, 2006). In particular, Rho GTPases Cdc42 and Rac1 control cell plasticity by initiating actin polymerization via *n*ucleation *p*romoting *f*actors (NPFs) that activate the ubiquitous Arp2/3 complex. The best-characterized NPFs N-WASP (*n*eural *W*iskott-*A*ldrich *s*yndrome *p*rotein) and WAVE (*WA*SP family *Ve*roprolin homolog) (Campellone and Welch, 2010) associate with phospholipid membranes, where they play pivotal roles in Arp2/3-dependent actin polymerization (Lebensohn and Kirschner, 2009; Machesky et al., 1999; Rohatgi et al., 2000). N-WASP is activated by Cdc42 to allow formation of cytoskeletal structures termed filopodia (Miki et al., 1998a; Rohatgi et al., 2000). WAVE is part of the heteropentameric WRC (composed of Abi, Cyfip, Nap1, and HSPC300 or their homologs) thought to be activated by Rac1 for generation of membrane ruffles and macropinocytosis (Gautreau et al., 2004; Miki et al., 1998b). While Rac1 can activate immunopurified WRC in buffer (Lebensohn and Kirschner, 2009), it has a very low affinity for WRC, suggesting the involvement of an additional unknown factor at the cell membrane (Chen et al., 2010; Davidson and Insall, 2011). By developing an assay where phospholipid-coated beads were used to track actin assembly machineries at the membrane, we uncovered that Rac1 is indeed insufficient for WRC activation in cell extract; this requires an Arf family GTPase (Koronakis et al., 2011). WRC recruitment and its activation at the membrane needed direct binding by an Arf GTPase which cooperates with Rac1 to elicit actin polymerization.

*Salmonella enterica* cause human and animal disease ranging from gasteroentiritis to typhoid fever. Central to bacterial infection is the invasion of nonphagocytic intestinal epithelial cells by pathogen-induced membrane ruffling and macropinocytosis (Galán, 2001). While it is clear that WRC is recruited to sites of forced cell entry, and that both membrane ruffling and subsequent pathogen macropinocytosis require the WRC (Hänisch et al., 2010; Shi et al., 2005; Unsworth et al., 2004), how *Salmonella* recruits and activates the WRC at the cell membrane is not known. *Salmonella* invasion is induced by delivery of virulence effector proteins into host cells. These include the effector SopE which localizes to the host plasma membrane (Cain et al., 2004), where it functionally mimics host GEFs by triggering release of GDP, to enable GTP-binding and activation of both Rac1 and Cdc42 (Hardt et al., 1998). SopE is sufficient for membrane ruffling and invasion (Misselwitz et al., 2011). Together, this suggested a potential role for SopE in bacterial manipulation of WRC. To investigate this possibility, we first examined the ability of SopE to recruit and activate the WRC at the membrane.

## Results

### Pathogen GEF SopE Triggers Recruitment of N-WASP and WRC

**Figure 1:**
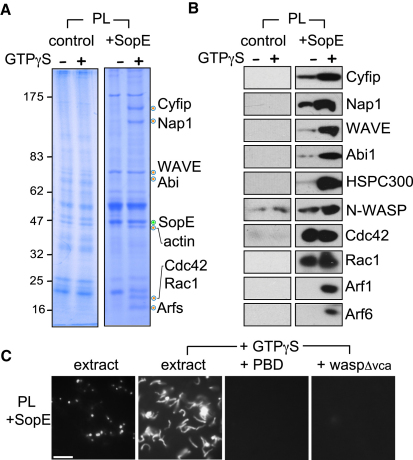
WRC and N-WASP Recruitment and Activation by SopE (A) Proteins recruited from extract to phospholipid bilayers by SopE. Silica beads coated with phospholipid bilayers (PL) alone (control) or decorated with SopE (PL +SopE) isolated from brain extract in the presence or absence of GTPγS. Recruited proteins were extracted, analyzed by SDS-PAGE and Coomassie blue staining. Proteins from gel bands marked with orange circles were identified (labeled right) by mass spectrometry. Membrane-bound SopE (green circle). Molecular weight markers in kDa (left). (B) Parallel immunoblotting of samples from (A) with indicated antibodies (right). See also Table S1. (C) Fluorescence microscopy of rhodamine-actin assembly on PL beads decorated with SopE (PL +SopE) in extract alone or supplemented with GTPγS, and in extract preincubated with inhibitors PBD and waspΔvca. Scale bar 15 μm.

Since the WRC exerts its NPF activity at cell membranes (Campellone and Welch, 2010), we first sought to investigate whether SopE could trigger recruitment of the WRC from brain cell extract to the membrane. The extract was prepared as recently described (Koronakis et al., 2011), and experiments were performed in the presence or absence of GTPγS (a nonhydrolyzable analog of GTP), which sustains GTPase activation. Purified SopE was bound onto silica beads coated with a phospholipid bilayer (PL beads) composed of equal amounts of phosphatidylcholine and phosphatidylinositol to generate SopE-decorated PL beads. PL beads alone (control) and SopE-decorated PL beads were each incubated in extract. The beads were isolated from the extract and extensively washed, and recruited proteins extracted from the membrane were then analyzed by Coomassie blue-stained SDS-PAGE (Figure 1A). In the absence of GTPγS (-), no significant differences were observed between control and SopE-decorated PL beads (+SopE) but when GTPγS (+) was added to the extract, SopE (marked by green circle) recruited additional proteins (orange circles) that were identified by mass spectrometry (Figure 1A and Table S1). The NPFs N-WASP and WAVE were identified, as were WRC components Cyfip, Nap1, and Abi. Rho GTPases Cdc42 and Rac1 were also evident on the SopE-decorated PL beads. Immunoblotting of the extracted proteins using commercially available antibodies confirmed SopE-dependent recruitment of small GTPases Cdc42 and Rac, and of NPFs N-WASP and WRC, including the smaller WRC constituent HSPC300 (Figure 1B). Immunoblotting also revealed that SopE (+SopE) recruited low levels of N-WASP and WRC in the absence of GTPγS (-), but this was substantially enhanced when GTPγS was added to the extract (+).

### SopE Triggers Activation of N-WASP but Not WRC

We next assessed whether membrane-bound SopE could activate the recruited NPFs. We have previously reconstituted N-WASP- and WRC-dependent actin assembly in brain cell extract so that NPF activity generated actin comet tails on PL beads that were consequently propelled through the extract (Koronakis et al., 2011). Here, when we added SopE-decorated PL beads (PL+SopE) to the extract (Figure 1C, extract), the beads recruited actin, and when GTPγS was added to the extract, comet tails were generated resulting in bead motility (extract +GTPγS). In contrast, control PL beads (i.e., without SopE) neither recruited actin nor generated comet tails, even with added GTPγS (data not shown). Comet tail formation and consequent motility of SopE-decorated PL beads was inhibited in extract preincubated with either PBD (GTPase-binding domain of PAK1 that inhibits Rac1 and Cdc42) (Benard et al., 1999) or waspΔvca (an N-WASP inhibitor) (Koronakis et al., 2011). These experiments demonstrate that SopE-dependent motility requires Rho GTPases and N-WASP. Therefore, while SopE recruited both N-WASP and WRC, only N-WASP was activated.

### Pathogen GEF SopE and Active Host Arf1 Cooperate to Activate WRC

**Figure 2:**
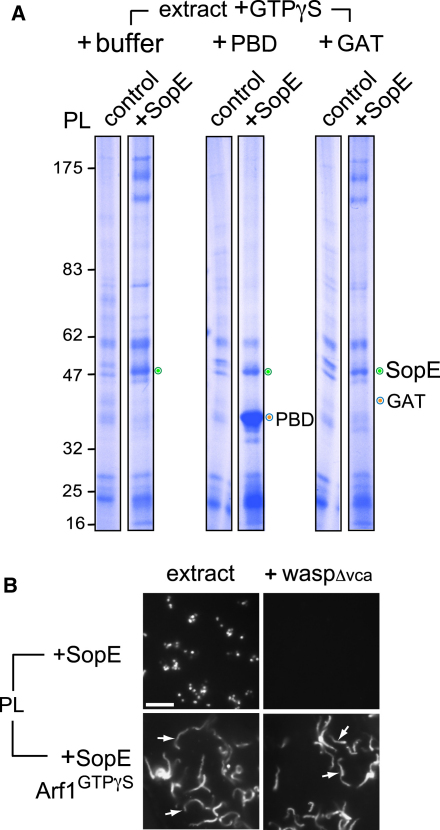
SopE Cooperation with Arf GTPase in Activation of WRC (A) Recruitment of Rho and Arf GTPase activation probes to SopE-decorated PL beads. PL beads alone (control) or decorated with SopE (PL +SopE) isolated from brain extract with GTPγS and from extract preincubated with control buffer, PBD, or GAT (orange circles). Membrane-bound SopE (green circle). Molecular weight markers in kDa (left). (B) Fluorescence microscopy of rhodamine-actin assembly on the PL beads decorated with SopE alone or together with anchored active myristoylated Arf1^GTPγS^ in extract with or without waspΔvca. Scale bar 15 μm. See also Figure S1.

We have established that WRC activation requires an Arf GTPase (Koronakis et al., 2011). Here, Arf GTPases were identified by mass spectrometry on SopE-decorated PL beads, and their recruitment in the presence of GTPγS was confirmed by immunoblotting (Figure 1B and Table S1). Since SopE did not lead to WRC activation (Figure 1C, +waspΔvca), it was possible that the recruited Arf GTPases were not active. To test this we exploited the ability of PBD and GAT (GGA domain that binds active Arf family GTPases) (Dell'Angelica et al., 2000) to specifically bind GTP-bound Rac1/Cdc42 and Arf GTPases, respectively. To track GTPase activation on the PL beads, extract containing GTPγS was preincubated with added buffer as control, purified PBD, or GAT, before parallel addition of control or SopE-decorated PL beads (+SopE) (Figure 2A). While SopE specifically recruited PBD, neither control nor SopE-decorated PL beads recruited GAT, which appeared indistinguishable from control lanes (+buffer), further confirming that SopE activates Rho but not Arf GTPases in the extract.

Since Arf GTPases appear inactive on SopE-decorated PL beads (Figure 2A) and motility of these beads was blocked by waspΔvca (Figure 2B, upper panel), we examined whether an active Arf would enable motility of SopE-decorated PL beads in N-WASP-inhibited extract (i.e., via WRC) (Koronakis et al., 2011). Purified, in vitro-myristoylated Arf1 was activated by loading with GTPγS and then anchored to PL beads. When the Arfl^GTPγS^ PL beads were decorated with SopE and added to the extract, long (∼14 μm) actin comet tails (arrows) formed, even in the absence of free-GTPγS, and the bead motility was unaffected by the presence of waspΔvca, demonstrating robust WRC activation (Figure 2B, lower panels). In contrast, Arfl^GTPγS^ PL beads alone (i.e., without SopE) recruited actin but only generated short (∼5 μm) actin comet tails (arrows) in N-WASP-inhibited extract, indicating much weaker WRC activation (Figure S1; Koronakis et al., 2011). These results show that an active Arf GTPase enables SopE activation of WRC.

### *Salmonella* Activates Arf1 for Pathogen Invasion of Host Cells

**Figure 3:**
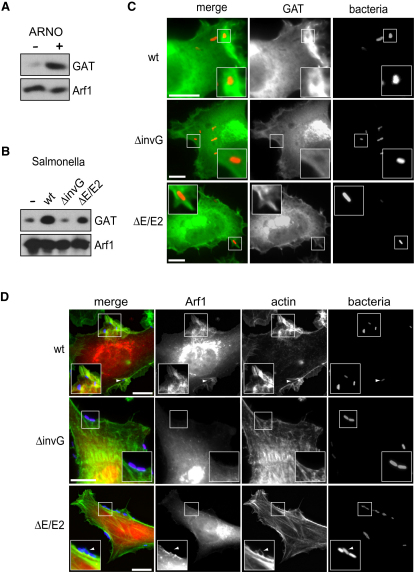
*Salmonella* Activation of Arf1 during Invasion (A and B) Activation of Arf1 by *Salmonella*. HeLa cells expressing Arf1^HA^ (Arf1) were lysed with detergent and the soluble fractions were incubated with ^GST^GAT (GAT), then Arf1^HA^ was immunoprecipitated. The fraction of GTP-bound Arf1^HA^ was determined by immunoblotting for coprecipitated GAT with anti-GST antibody and anti-HA as control. In (A), HeLa cells expressing Arf1^HA^ alone (-) or together with ARNO (+) are shown. (B) shows HeLa cells expressing Arf1^HA^ either noninfected (-) or infected (15 min) with wild-type (WT), *ΔinvG*, or *ΔE/E2* strains of *Salmonella*. (C and D) Localization of ^CFP^GAT (C) or Arf1^RFP^ (D) in HeLa cells infected for 15 min with wild-type (WT), *ΔinvG*, or *ΔE/E2* strains of *Salmonella* (bacteria). Alexa Fluor 488-phalloidin was used to visualize actin in (C). Insets show magnified area. Scale bar 8 μm.

As WRC is key to *Salmonella* invasion by macropinocytosis (Hänisch et al., 2010; Shi et al., 2005; Unsworth et al., 2004), we investigated the putative role of Arf1 in this process. Initially, we examined the activation status of Arf1 during invasion using a GAT assay. In this assay, HeLa cells expressing HA-tagged Arf1 were lysed with detergent before incubation with purified GAT that binds GTP-bound Arf1. Next, Arf1 was immunoprecipitated and assessed for coprecipitated GAT by immunoblotting (Figure 3A). In a control experiment, GAT was coprecipitated from HeLa cells expressing Arf1 alone (-), but when Arf1 was coexpressed with host ARNO (+), an Arf GEF used as a tool to activate Arf1, coprecipitation of GAT was increased (Figure 3A), confirming Arf1 activation. In parallel, HeLa cells were infected with either wild-type (WT) *Salmonella enterica* Typhimurium (henceforth *Salmonella*) or a noninvasive strain carrying an *invG* null mutation making it incapable of delivering effectors (Figure 3B). While no significant differences were observed when GAT was coprecipitated from either noninfected control cells (-) or *ΔinvG*-infected cells, WT infection increased coprecipitation of GAT, demonstrating effector-driven activation of Arf1 during invasion. In Figure 2A, we showed that SopE was incapable of activating Arf GTPase in extract. To confirm that this is also the case during *Salmonella* invasion, we performed a GAT assay with cells infected with *Salmonella* carrying a null mutation in *sopE* and its close homolog *sopE2* (*ΔE/E2*). Indeed, only a small reduction in coprecipitated GAT was observed in *ΔE/E2*-infected cells relative to WT-infected cells, showing that Arf1 activation during invasion is independent of *Salmonella* GEFs and is instead mediated via a distinct bacterial effector(s).

Arf1 activation by *Salmonella* prompted us to examine the localization of the active Arf during *Salmonella* invasion by infecting HeLa cells expressing ^CFP^GAT with either WT, *ΔinvG*, or *ΔE/E2 Salmonella* (Figure 3C). Fluorescence microscopy of the infected cells showed that GAT was enriched at the perinuclear Golgi apparatus (where the functions of active Arf1 are best characterized) and at the plasma membrane. While GAT did not localize to *ΔinvG* mutant *Salmonella*, GAT was observed accumulating at WT-triggered membrane ruffles during invasion (magnified insets). GAT was also observed beneath adherent *ΔE/E2*, verifying that Arf1 activation occurs independently of SopE during invasion. As GAT can bind multiple Arf isoforms (Nakayama and Takatsu, 2005), we sought to confirm that Arf1 localizes to membrane ruffles induced by *Salmonella* by performing the same infection with HeLa cells expressing Arf1^RFP^ (Figure 3D), which behaves indistinguishably from endogenous Arf1 in cells (Cohen et al., 2007). Arf1 was observed only at the Golgi in cells infected with the *ΔinvG* mutant, which induced no cytoskeletal rearrangements (magnified insets). In contrast, WT *Salmonella* triggered membrane ruffles that were enriched in Arf1 (magnified insets), which also surrounded invading bacteria (arrow and magnified insets). In *ΔE/E2*-infected cells, only modest cytoskeletal rearrangements were apparent beneath adherent *Salmonella* (Figure 3D, arrow within inset), likely due to the effectors SipA, SipC, and SopB that promote actin polymerization (McGhie et al., 2009). The characteristic membrane ruffles, though, were absent, which is consistent with the inability of *ΔE/E2* to activate Rac1 (Friebel et al., 2001; Patel and Galán, 2006). In contrast to *ΔinvG*, *ΔE/E2* recruited Arf1 to bacterial invasion sites (Figure 3D, inset), albeit to a lesser extent, suggesting an additional factor drives the Arf1 recruitment observed in WT-infected cells. Taken together, these results establish that *Salmonella* activates Arf1, but that its recruitment alone is not sufficient to induce membrane ruffling, as this also requires the action of SopE.

**Figure 4:**
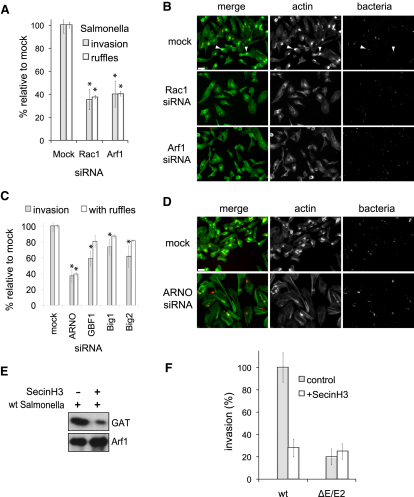
Host Arf GEF Promotion of *Salmonella*-Induced Ruffling and Invasion (A) Influence of Rac1 and Arf1 depletion on *Salmonella* invasion and ruffle formation. HeLa cells were transfected with either mock, Rac1, or Arf1 siRNA 72 hr before infection with WT *Salmonella* (15 min). To quantify *Salmonella* invasion, washed infected cells were incubated with gentamicin for 2 hr to kill extracellular bacteria, and invasion was quantified by colony counts. To quantify *Salmonella* with ruffles, fixed infected cells were stained with Alexa Fluor 488-phalloidin to visualize actin. The number of fluorescently labeled bacteria in each field of view was counted and the proportion of those bacteria associated with actin ruffles determined. Asterisks indicate a significant difference from mock (p < 0.05, ANOVA; n = 3). Error bars represent ± SEM. Knockdowns were quantified by qRT-PCR, data not shown. (B) Representative fluorescence images of *Salmonella*-induced ruffles in mock- and Arf1-depleted cells from (A). Scale bar 130 μm. Arrows indicate *Salmonella*-induced ruffles in mock. (C) Influence of Arf family GEF depletion on *Salmonella* invasion and ruffle formation. HeLa cells were transfected with either mock or indicated Arf GEF siRNA 72 hr before infection with WT *Salmonella* (15 min). Quantification of invasion and *Salmonella* with ruffles was performed as in (A). (D) Representative fluorescence images of *Salmonella*-induced ruffles in mock- and ARNO-depleted cells from (C). Scale bar 130 μm. Asterisks indicate a significant difference from mock (p < 0.05, ANOVA; n = 3). See also Figure S2. (E) ARNO activation of Arf1 during *Salmonella* invasion. HeLa cells expressing Arf1^HA^ (Arf1) were treated with either DMSO (control) or an inhibitor of the cytohesin family (SecinH3) 1 hr before infection (15 min) with WT *Salmonella*. Cells were lysed with detergent, and the soluble fractions were incubated with ^GST^GAT (GAT), then Arf1^HA^ was immunoprecipitated. The fraction of GTP-bound Arf1^HA^ was determined by immunoblotting for coprecipitated GAT with anti-GST antibody and anti-HA as control. (F) Influence of ARNO inhibition on *Salmonella* invasion. HeLa cells were treated with either DMSO (control) or an inhibitor of the cytohesin family (SecinH3) 1 hr before infection with WT or *ΔE/E2* strains of *Salmonella* (15 min), and quantification of *Salmonella* invasion was performed as in (A).

As SopE and Arf1 cooperate in the activation of the WRC (Figure 2B), it is possible that Arf1 is recruited by *Salmonella* to promote this synergy with SopE for triggering membrane ruffling and invasion. To examine this possibility, we depleted endogenous Arf1 by transfecting a pool of three specific siRNAs against Arf1 72 hr prior to infection with WT *Salmonella* and quantification of pathogen invasion (Figure 4A). Relative to mock-transfected control cells (i.e., cells transfected with negative control “Allstars” siRNA), *Salmonella* invasion was reduced by ∼60% in Arf1-depleted cells. A comparable reduction was observed when Arf1 was depleted with each siRNA individually (data not shown). The proportion of *Salmonella* associated with zones of actin-rich invasion ruffles (arrows) was reduced by ∼60% in Arf1-depleted cells relative to the mock, exactly mirroring the reduction seen in invasion (Figure 4A) and illustrated in Figure 4B. The decrease in invasion and ruffling in Arf1-depleted cells was comparable to that observed when HeLa cells were depleted of Rac1 (Figures 4A and 4B), a pivotal regulator of *Salmonella* invasion (Patel and Galán, 2006). These results show that *Salmonella* activates Arf1 to promote membrane ruffling and invasion.

### Host Arf GEF ARNO Promotes *Salmonella-*Induced Ruffling and Invasion

As *Salmonella* encodes no known Arf GEF, we reasoned that *Salmonella* might act by utilizing a host Arf GEF to activate Arf1. Arf1 GEFs can be divided into three subfamilies, namely GBF1, Big (isoforms Big1 and Big2), and cytohesin, of which ARNO (cytohesin2) is the best characterized (Donaldson and Jackson, 2011). These Arf GEFs were depleted individually by siRNA transfection of HeLa cells 72 hr before *Salmonella* infection and quantification of invasion (Figure 4C). Arf GEF depletion was verified by immunoblotting (Figure S2A). In all cases, Arf GEF depletion significantly inhibited *Salmonella* invasion, with the most substantial inhibition observed in ARNO-depleted cells, where it was reduced by ∼63% relative to mock siRNA-transfected control cells. While no significant difference was observed in *Salmonella*-induced ruffling when GBF1, Big1, and Big2 were depleted, generation of actin-rich ruffles was reduced by ∼60% in ARNO-depleted cells. This was illustrated by fluorescence microscopy where zones of enriched-actin corresponding to membrane ruffles triggered by the *Salmonella* (bacteria) in mock- but not ARNO-depleted cells (Figure 4D). To verify that ARNO activates Arf1 during *Salmonella* invasion, we used the GAT assay to examine the levels of GTP-bound Arf1 in the presence of SecinH3 (Figure 4E), a specific inhibitor of the cytohesin family (Hafner et al., 2006). GAT was coprecipitated with immunoprecipitated Arf1 in *Salmonella*-infected control cells (-), reconfirming robust Arf1 activation, but this was substantially reduced in the presence of SecinH3 (+), establishing that ARNO activates Arf1 during pathogen invasion.

Since both ARNO (Figure 4C) and SopE are required for generating membrane ruffles (Figure 3D), we sought to confirm that ARNO acted with SopE to promote invasion by incubating HeLa cells with SecinH3 before infection with WT and *ΔE/E2 Salmonella* (Figure 4F). Invasion of WT *Salmonella* was significantly reduced by ∼73% in the presence of SecinH3, confirming ARNO promotion of pathogen uptake. This reduction was mirrored in control cells infected with *ΔE/E2* (∼80%) demonstrating that blockade of SopE or ARNO signaling impairs invasion to the same extent. When SecinH3-treated cells were infected with *ΔE/E2,* invasion was inhibited (∼75%), revealing no additive effect to that seen in the absence of SecinH3. These results reveal that SopE and ARNO trigger membrane ruffling and invasion via the same route.

In addition to Arf1, the mammalian Arf GTPase family includes members Arf3, Arf4, Arf5, and Arf6 (Donaldson and Jackson, 2011). Since ARNO has been shown to activate Arf3 and Arf6 in vitro (Frank et al., 1998; Meacci et al., 1997), we examined whether any additional Arf GTPases promote invasion by depleting Arf3, Arf4, Arf5, and Arf6 individually by siRNA transfection of HeLa cells 72 hr before infection with WT *Salmonella* (Figure S2B). While invasion was not significantly affected by Arf4 and Arf5 depletion, invasion was reduced in Arf3-depleted cells (∼50%) and Arf6-depleted cells (∼40%), indicating that ARNO triggers invasion via Arf1, Arf3, and Arf6.

### *Salmonella* Recruits ARNO via SopB-Induced PIP3 Production and Arf6

**Figure 5:**
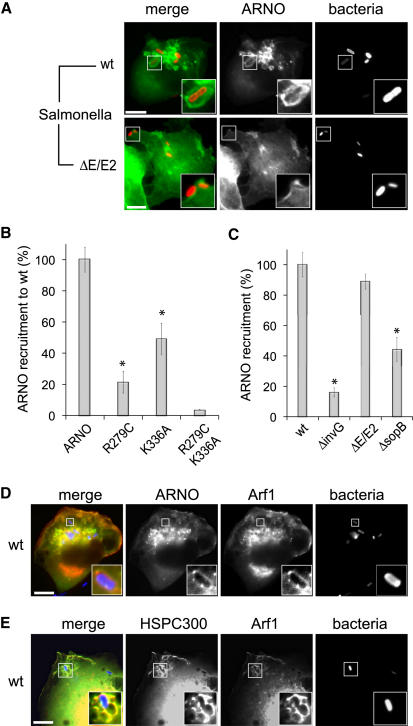
ARNO Generation of *Salmonella*-Induced Macropinosomes (A) Localization of ARNO during *Salmonella* invasion. HeLa cells expressing ^CFP^ARNO (ARNO) were infected for 15 min with fluorescently labeled WT or *ΔE/E2 Salmonella* (bacteria) as indicated (left). Scale bar 8 μm. Insets show magnified area. (B) ARNO recruitment by *Salmonella*. To quantify ARNO recruitment, HeLa cells expressing either ^CFP^ARNO (ARNO), ^CFP^ARNO^R279C^ (R279C), ^CFP^ARNO^K336A^ (K336A), or ^CFP^ARNO^R279CK336A^ (R279C K336A) were infected with fluorescently labeled WT *Salmonella*, and the proportion of bacteria enriched with ARNO was determined microscopically. Asterisks indicate a significant difference from mock (p < 0.05, ANOVA; n = 3). Error bars represent ± SEM. (C) Quantification of ^CFP^ARNO (ARNO) recruitment by WT, *ΔinvG*, *ΔE/E2*, and *ΔsopB Salmonella* strains was performed as in (B). See also Figure S3. (D and E) Localization of ARNO, Arf1, and HSPC300 during *Salmonella* invasion. HeLa cells expressing ^CFP^ARNO and Arf1^RFP^ (Arf1) (D) or ^CFP^HSPC300 (HSPC300) and Arf1^RFP^ (E) were infected as (A).

Since membrane-ruffling requires the WRC (Hänisch et al., 2010) and our data show that ARNO specifically promotes this pathogen-induced ruffling and invasion, we used fluorescence microscopy to examine ARNO localization in HeLa cells expressing ^CFP^ARNO (ARNO) infected with WT or *ΔE/E2 Salmonella* (Figure 5A). Profuse macropinosomes enriched in ARNO, often containing engulfed *Salmonella* (magnified insets), were generated at *Salmonella* invasion foci in a SopE/E2-dependent manner (macropinosomes were observed in ∼72% of WT-infected cells, compared to only ∼10% in *ΔE/E2*-infected cells and ∼4% in *ΔinvG*-infected cells, shown in Figure S3A). These ARNO-enriched pathogen-containing macropinosomes colocalized with polymerized actin (Figure S3B). To confirm that it was the GEF activity of ARNO which promoted macropinosome formation, HeLa cells expressing a catalytically attenuated (i.e., reduced by 99.74%) ARNO variant (ARNO^E156D^) (Béraud-Dufour et al., 1998) were infected with WT *Salmonella* (Figure S3C). While ARNO^E156D^ was still recruited to *Salmonella* invasion sites, macropinosomes were not induced.

The localization of ARNO^E156D^ at *Salmonella* invasion sites showed that recruitment of ARNO was independent of GEF activity. ARNO contains an N-terminal catalytic Sec7 domain followed by a linker to the C terminus comprising a *p*leckstrin *h*omology (PH) domain and a polybasic motif (Donaldson and Jackson, 2011). ARNO recruitment to the plasma membrane depends upon its PH domain, which binds the phosphoinositide PIP3 and Arf6 (Cohen et al., 2007; Klarlund et al., 2000; Macia et al., 2000). To elucidate how *Salmonella* recruits ARNO, we investigated the role of PIP3 and Arf6 by examining WT-infected HeLa cells expressing either ARNO^R279C^ (an ARNO derivative defective in binding PIP3) (Venkateswarlu and Cullen, 2000) or ARNO^K336A^ (an ARNO derivative defective in binding Arf6) (Stalder et al., 2011), using fluorescence microscopy. Relative to ARNO (which was observed enriched around ∼64% of WT *Salmonella*), recruitment of ARNO^R279C^ was reduced by ∼79%, while ARNO^K336A^ was reduced by ∼46% (Figure 5B). No recruitment of ARNO^R279C/K336A^ (ARNO incapable of binding both PIP3 and Arf6) was observed, showing that PIP3 and, to a lesser extent, Arf6 are responsible for recruiting ARNO to *Salmonella*.

Since Arf1 activation requires *Salmonella* effectors (Figure 3B), we reasoned that they might facilitate Arf1 activation by recruiting ARNO to invasion sites. Indeed, ARNO recruitment was reduced by ∼84% in *ΔinvG*-infected cells relative to WT-infected cells (Figure 5C). Consistent with SopE-independent activation of Arf1 (Figure 3A), no significant difference in ARNO recruitment by WT and *ΔE/E2 Salmonella* was observed (Figure 5C). As PIP3 binding was crucial to ARNO recruitment **(**Figure 5B**)** and the *Salmonella* effector SopB is known to trigger generation of PIP3 at invasion ruffles (Mallo et al., 2008), we next examined its role in ARNO recruitment by infecting HeLa cells with *ΔsopB Salmonella*. ARNO recruitment was reduced by ∼56%, confirming a key role for SopB (Figure 5C). These results show that *Salmonella* recruits ARNO via its PH domain through SopB-induced PIP3 production and Arf6.

### ARNO Activates Arf GTPases to Recruit WRC to the Membrane

When Arf1^RFP^ was coexpressed with ^CFP^ARNO or ^CFP^GBF1, Arf1 colocalized with each GEF, but only ARNO recruited Arf1 to the plasma membrane (Figure S3D). This is consistent with SopE-independent Arf1 recruitment (Figure 3D) and agrees with ARNO regulating Arf1 at the plasma membrane, while GEFs like GBF1, Big1, and Big2 activate Arf1 at the Golgi (Donaldson and Jackson, 2011). Since ARNO recruited Arf1 to the plasma membrane, and Arf at the membrane was crucial to WRC activation and subsequent actin assembly (Figure 2B), we hypothesized that ARNO, Arf1, and the WRC might colocalize at WT *Salmonella*-induced macropinosomes. Fluorescence microscopy of infected HeLa cells expressing Arf1^RFP^ together with either ^CFP^ARNO (Figure 5D) or the WRC component ^CFP^HSPC300 (Figure 5E) showed that Arf1 indeed colocalizes with both ARNO and HSPC300 at *Salmonella*-induced macropinosomes (∼92% and ∼86%, respectively). These results show that ARNO promotes effector-driven formation of macropinosomes enriched in Arf1, actin, and HSPC300.

**Figure 6:**
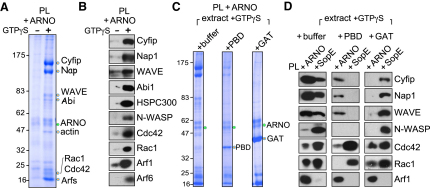
WRC Recruitment by ARNO (A) Proteins recruited from extract to phospholipid bilayers by ARNO. PL beads decorated with ARNO (PL +ARNO) isolated from brain extract in the presence or absence of GTPγS. Recruited proteins were extracted, analyzed by SDS-PAGE and Coomassie blue staining. Proteins from gel bands marked with orange circles were identified (labeled right) by mass spectrometry. Membrane-bound ARNO (green circle). Molecular weight markers in kDa (left). (B) Parallel immunoblotting of samples from (A) with indicated antibodies (right). (C) Proteins recruited to PL beads by ARNO from extract containing Rho and Arf GTPase activation probes. PL beads decorated with ARNO (PL +ARNO) isolated from brain extract supplemented with GTPγS and from extract preincubated with control buffer, PBD, or GAT (orange circles). Membrane-bound ARNO (green circle). Molecular weight markers in kDa (left). (D) PL beads decorated with either ARNO (samples from C) or SopE (Figure 2A) were analyzed by SDS-PAGE and immunoblotting with indicated antibodies (right). See also Figure S4.

The colocalization of Arf1 and WRC at ARNO-enriched, pathogen-induced macropinsomes prompted us to test whether ARNO could trigger recruitment of the WRC to the membrane from brain cell extract. PL beads decorated with purified ARNO (green circle) were incubated in extract with and without added GTPγS (Figure 6A). Several distinct proteins were recruited by ARNO-decorated PL beads in a GTPγS-dependent manner (orange circles). These were identified by mass spectrometry as WRC components Cyfip, Nap1, WAVE, and Abi, and Rho GTPases Cdc42 and Rac1 (Figure 6A and Table S2). A low molecular weight protein band of particular prominence contained Arf GTPases. Immunoblotting of the extracted proteins confirmed ARNO-dependent recruitment (+ARNO) of WRC components, including HSPC300, and small GTPases Cdc42, Rac1, Arf1, and Arf6 (Figure 6B). Immunoblotting also revealed that ARNO recruited low levels of WRC in the absence of GTPγS (-), but recruitment was considerably enhanced when GTPγS was added to the extract (+).

As we had established that SopE recruited the WRC by activating a Rho GTPase, most likely Rac1 (Figures 1A and 2A), we set out to ascertain whether WRC recruitment by ARNO was achieved through Rho or Arf GTPase activation. ARNO-decorated PL beads were added to extract containing GTPγS, preincubated with added buffer as control, purified PBD, or GAT (Figure 6C). ARNO-decorated PL beads recruited PBD (orange circle), which was absent in the control lane (+buffer) (Figure 6C). It was noticeable that less PBD was recruited by ARNO-decorated PL beads (Figure 6C) relative to SopE-decorated PL beads shown in Figure 2A (PBD), indicating much weaker activation of Rho GTPases by ARNO. GAT (orange circle) was recruited in abundance by ARNO, demonstrating strong Arf GTPase activation (Figure 6C).

As PBD binds Rac-GTP and GAT binds Arf-GTP, we examined whether their association with PL beads decorated with either SopE (samples from Figure 2A) or ARNO (Figure 6C) inhibited recruitment of WRC (Figure 6D). Immunoblotting showed that control buffer had no effect on WRC recruitment by PL beads decorated with either SopE or ARNO. In contrast, WRC recruitment via ARNO was blocked by GAT, but not PBD, while SopE recruitment of the WRC was abolished by PBD, but unaffected by GAT. These results show that WRC recruitment via ARNO requires an Arf GTPase, while recruitment via SopE requires a Rho GTPase. In addition to WRC, PBD blocked Arf1 recruitment by SopE showing that Rho GTPase activation promotes recruitment of Arf1. This suggests that SopE recruits inactive Arf GTPases (Figures 1A and 2A and Table S1) to the membrane via their direct interaction with the WRC (Koronakis et al., 2011) rather than by direct interaction with SopE.

### Pathogen SopE and Host ARNO Cooperate to Recruit and Activate WRC

We have shown that SopE mediated recruitment, but not activation of WRC (Figure 1); this was triggered by Arf1 (Figure 2), which in turn is recruited and activated by ARNO (Figures 4, 5, and 6). As SopE and ARNO promoted macropinocytosis of *Salmonella* (Figures 3, 4, and 6), a process requiring WRC (Hänisch et al., 2010; Shi et al., 2005; Unsworth et al., 2004), we assessed whether SopE and ARNO cooperate in recruitment and activation of the WRC.

**Figure 7:**
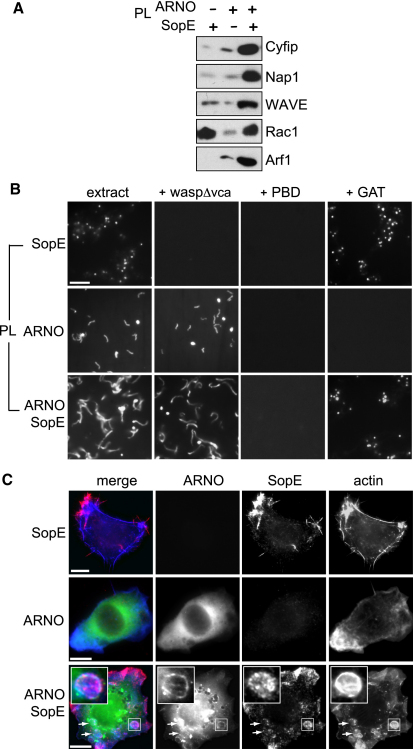
Cooperation of SopE and ARNO in Recruitment and Activation of WRC (A) WRC recruitment to phospholipid bilayers by ARNO and SopE together. PL beads decorated with either ARNO or SopE, or both GEFs together were isolated from brain extract in the absence of GTPγS. Recruited proteins were extracted before analysis by SDS-PAGE and immunoblotting with indicated antibodies (right). See also Figure S4. (B) Fluorescence microscopy of rhodamine-actin assembly on PL beads from (A) in extract without GTPγS in the presence or absence of waspΔvca, PBD, or GAT. Scale bar 15 μm. (C) Actin assembly by ARNO and SopE in cells. HeLa cells expressing either SopE^FLAG^ (SopE) or ^CFP^ARNO (ARNO) or both GEFs together were fixed, then stained with anti-FLAG to label SopE^FLAG^ and Alexa Fluor 350-phalloidin to label actin. Insets magnify SopE, ARNO, and actin colocalizing at macropinosomes. Scale bar 8 μm.

PL beads decorated with either SopE or ARNO, or both GEFs together, were added to extract in the absence of GTPγS, and then the recruitment of WRC components and small GTPases Arf1 and Rac1 were investigated by immunoblotting (Figure 7A). In all cases, the WRC was recruited, but this was substantially enhanced when both ARNO and SopE were together. Consistent with this observation, recruitment of WRC activators and direct binding partners, Arf1 and Rac1, was also increased by the presence of both SopE and ARNO. The substantial increase in Arf1 recruitment when SopE and ARNO were together (Figure 7A) was also observed during invasion (Arf1 was more enriched around WT bacteria than *ΔE/E2*, as seen in Figure 3D). To examine this phenomenon in more detail, PL beads decorated with either ARNO or ARNO^E156D^ (E156D), a derivative with attenuated GEF activity, were added to the extract in the presence of GTPγS, and then the recruitment of Arf1 was determined by immunoblotting (Figure S4). ARNO but not ARNO^E156D^ triggered Arf1 recruitment, confirming that ARNO GEF activity brings Arf1 to the membrane. When SopE was added to either ARNO or ARNO^E156D^ PL beads, recruitment of Arf1 was enhanced, which was now also detectable on ARNO^E156D^ PL beads. Consistent with data in Figure 6D, this promotion was blocked by PBD, indicating that SopE enhances Arf1 recruitment via Rho GTPases. Although it remains possible that SopE stimulates GEF activity of ARNO and ARNO^E156D^ (Figure S4), the enhanced Arf1 recruitment when SopE and ARNO were together (Figure 7A) appears most likely due to increased amounts of the Arf1 binding partner WRC, which may stabilize active small GTPases at the membrane.

We next examined WRC activation on PL beads decorated with either SopE or ARNO, or both GEFs together, in extract without GTPγS by fluorescence microscopy (Figure 7B). As shown in Figure 1C, no activation of WRC was observed on SopE-decorated PL beads in the presence of waspΔvca (Figure 7B, top panel). In contrast, ARNO-decorated PL beads generated actin comet tails that were still formed even in extract preincubated with waspΔvca demonstrating WRC activation (middle panel). Furthermore, ARNO-dependent comet tail formation was blocked by both PBD and GAT individually, confirming WRC activation required both Rho and Arf GTPases (middle panel). We noticed that ARNO-decorated PL beads generated short comet tails that were reminiscent of PL beads with anchored Arf1^GTPγS^ (Figure S1; Koronakis et al., 2011), perhaps indicating insufficient levels of active Rac1. This appeared to be the case, as actin assembly by PL beads decorated with ARNO and SopE together was enhanced so that long comet tails were formed, even in the presence of waspΔvca (bottom panels), demonstrating robust WRC activation. This WRC activation was inhibited by GAT as the beads recruited actin but failed to form comet tails showing that active Arf GTPases are required (bottom panels). This weaker actin assembly in the presence of GAT by PL beads decorated with both ARNO and SopE was mediated via the SopE-triggered Cdc42/N-WASP pathway, as SopE-decorated PL beads (top panels) also recruited actin in the presence of GAT, but not waspΔvca or PBD. PBD abolished actin assembly by PL beads decorated with both ARNO and SopE (bottom panels). These results show that SopE and ARNO cooperate to recruit and activate the WRC for actin assembly.

To confirm SopE and ARNO cooperate in cells to trigger actin assembly, the actin cytoskeleton was examined in HeLa cells expressing either SopE^FLAG^ or ^CFP^ARNO alone, or both GEFs in combination (Figure 7C). Actin-rich macropinosomes analogous to those formed during *Salmonella* infection (Figures 5A and S3B) were generated when ARNO and SopE were expressed together (exemplified by magnified insets and the additional examples highlighted with arrows) but not individually, demonstrating their cooperation during actin assembly in cells.

## Discussion

Establishing how the cell coordinates WRC recruitment and activation is crucial to understanding actin polymerization at the membrane during processes such as cell motility, membrane ruffling, and pathogen invasion. *Salmonella* is known to activate Rho GTPases for membrane ruffling during invasion (Patel and Galán, 2006), but while this cytoskeletal remodelling requires WRC (Hänisch et al., 2010), it was not known how *Salmonella* regulates such NPF activity. In this study, we have shown that the *Salmonella* GEF SopE is able to recruit and activate Cdc42 and Rac1 at the membrane, leading to recruitment of N-WASP and WRC. NPF recruitment and their activation were separable, as SopE triggered recruitment of N-WASP and WRC but only N-WASP was activated. This study of pathogen subversion builds on our recent demonstration that active Rac1 is not sufficient for WRC activation, but also requires direct binding by an active Arf GTPase (Koronakis et al., 2011). Here, recombinant active Arf1 activated the WRC that was recruited by SopE, indicating a possible role for Arf GTPases in WRC signaling during *Salmonella* invasion. This proved to be the case, as *Salmonella* activated Arf1, which localized to invasion foci, promoting both membrane ruffling and pathogen macropinocytosis. A role for active Arf was further supported by an RNAi screen of host Arf GEFs, depletion of which inhibited pathogen invasion.

Bacterial pathogens are known to subvert small GTPase signaling by encoding functional mimics of their host GEF counterparts. For example, Arf GTPase is hijacked by the pathogen *Legionella pneumophila*, which delivers its own Arf1 GEF to promote Arf1 localization to the *Legionella*-containing vacuole during bacterial replication in host macrophages (Nagai et al., 2002). Intriguingly, *Salmonella* encodes no known Arf GEF, so its activation of Arf1 suggested that pathogen invasion might be facilitated by utilization of a host Arf GEF. The RNAi screen revealed a particular importance for the Arf GEF ARNO in promoting *Salmonella*-induced ruffling and macropinocytosis, and consistent with this, *Salmonella* invasion was impaired when cells were treated with a chemical inhibitor of ARNO. ARNO promotion of invasion via Arf1 was supported by its ability to recruit and activate Arf1 at the plasma membrane, as this process is known to promote generation of membrane ruffles and macropinosomes (Cohen et al., 2007; Stalder et al., 2011). Sure enough, expression of wild-type but not catalytically inactive ARNO enabled *Salmonella* induction of profuse macropinosomes in a SopE-dependent manner that colocalized with actin and engulfed bacteria. The crucial role for ARNO in SopE-driven pathogen macropinocytosis was emphasized by the SopB-mediated recruitment of ARNO (Figure S5A) and enforces the view that *Salmonella* effectors act in concert to penetrate host cells.

It is known that ARNO catalyzes nucleotide exchange most effectively on Arf1 both in cells and in vitro (Cohen et al., 2007; Macia et al., 2001), which is consistent with impaired *Salmonella* invasion following Arf1 depletion (Figure 4A). Nonetheless, nucleotide exchange by ARNO in vitro has also been observed on Arf3 and Arf6 (Frank et al., 1998; Meacci et al., 1997) which opened up the possibility that additional isoforms of the 29-member Arf family may also play a role during *Salmonella* invasion. This is indeed the case as both Arf3 and Arf6 enhanced *Salmonella* invasion. Consistent with this, we recently established that multiple members of the Arf family can recruit and activate the WRC (Koronakis et al., 2011), so this redundancy is likely exploited by *Salmonella* to activate WRC. A wider involvement of Arfs in this path is also possible, given that they act upstream to promote ARNO-mediated Arf1 activation (Donaldson and Jackson, 2011). For example, ARNO is recruited to the plasma membrane by GTP-bound Arf6 (Cohen et al., 2007) that binds the ARNO PH domain to facilitate Arf1 activation, which itself triggers a positive feedback effect on ARNO for further exchange activity (DiNitto et al., 2007; Stalder et al., 2011). This mechanism seems to operate during invasion, as we show that Arf6 promoted recruitment of ARNO to *Salmonella* entry sites, indicating how Arf6 enhances pathogen invasion (depicted in our model in Figure S5A).

WRC activation is achieved by concomitant activation of Arf and Rac GTPase at the membrane (Koronakis et al., 2011). This study points to a complex regulatory network of pathogen- and host-encoded proteins cooperating to facilitate *Salmonella* macropinocytosis via WRC. We observed *Salmonella*-induced macropinosomes enriched with ARNO, Arf1, and WRC component HSPC300. This signaling platform was recapitulated in vitro, where ARNO triggered Arf GTPase-dependent actin polymerization at the membrane by recruitment and activation of the WRC. However, ARNO, in isolation, only generated short actin comet tails, owing to insufficient levels of active Rac1. During *Salmonella* invasion, SopE is known to localize to the plasma membrane (Cain et al., 2004) and would therefore be ideally placed to cooperate with ARNO by activating Rac1, thus achieving WRC activation. We showed that SopE and ARNO were both required for generating *Salmonella*-induced macropinosomes and that they facilitated pathogen invasion via the same route, supporting their dual role in WRC activation. Indeed, we showed that WRC recruitment to the membrane, its activation, and subsequent actin polymerization was enhanced when SopE and ARNO cooperated, a synergy that was also evident when SopE and ARNO were coexpressed in cells. Our data provide a mechanism in which cooperating GEFs trigger coincident activation of Arf and Rac at specific locations to activate the WRC and initiate actin polymerization (illustrated in our model in Figure S5B). We propose that *Salmonella* employs this mechanism by recruiting the host GEF ARNO and delivering the bacterial GEF SopE to the plasma membrane beneath the invading bacterium, which triggers WRC-dependent membrane ruffling and consequent pathogen macropinocytosis.

## Experimental Procedures

### Bacterial Strains and Infection of HeLa cells

Wild-type *Salmonella enterica* serovar Typhimurium SL1344 (gift from Jean Guard-Petter), isogenic *ΔsopEΔsopE2* (kind gift from Wolf-Dietrich Hardt), *ΔinvG*, and *ΔsopB* were constructed as described (Humphreys et al., 2009). For visualizing *Salmonella* by fluorescence microscopy, bacteria were washed with phosphate-buffered saline, conjugated to either Alexa Fluor 350 or Alexa Fluor 594 carboxylic acid succinimidyl ester (15 min, 37°C), washed in Tris (pH 7.4)-buffered saline, then used to infect HeLa cells (moi of 50). Infected HeLa cells (15 min, unless stated otherwise) were used either to quantify invasion by gentamicin protection (Humphreys et al., 2009) or to quantify *Salmonella*-induced membrane ruffling by determining the proportion of adherent *Salmonella* associated with actin-rich foci using fluorescence microscopy (∼150 cells per experiment). When appropriate, HeLa cells were incubated with 25 μM SecinH3 (Merck). Immunofluorescence microscopy and images were assembled as described (Humphreys et al., 2009). All experiments were performed at least three times. Geometric means were calculated and significance determined by one-way analysis of variance (ANOVA) followed by a post hoc Dunnett's comparison. p < 0.05 was considered significant.

### Actin-Based Motility by Phospholipid Beads

Preparation of porcine brain extract, actin-based motility by phospholipid-coated beads, and isolation of bead membrane-associated proteins have been described in detail (Koronakis et al., 2011). In brief, a 60 μl motility-mix (extract) was prepared on ice in the following order; 40 μl brain extract, 3 μl 20× energy mix (300 mM creatine phosphate, 40 mM MgCl2, 40 mM ATP), 3 μl G-actin/rhodamine actin (140 μM, prepared as described) (McGhie et al., 2004), 6 μl 10× salt buffer (600 mM KCL, 200 mM 3-phosphoglycerate), 6 μl 50 mM BAPTA (Merck) and 1 μl 300 mM DTT (Merck) and, when appropriate, 1 μl 30 mM GTPγS (Roche). Actin-motility assays were initiated by adding 0.1 vol phospholipids-coated beads to 10 μl motility mix, then1 μl was applied to a microscope slide. When indicated, extract was preincubated with the inhibitors recombinant GAT^165–314^, PAK-PBD, and waspΔvca, as described (Koronakis et al., 2011). For protein isolation, actin-motility assays were scaled up (1 ml) then incubated (20 min, RT) before phospholipid-coated beads were isolated by low-speed centrifugation (1000 g), washed ten times in HKSM (20 mM HEPES [pH7.4], 100 mM KCl, 5 mM MgCl_2_) buffer, and then proteins were extracted with SDS-UREA sample buffer.

### GAT Assay for Determining Cellular Level of Arf1-GTP

The methodology was adapted from Yoon et al., 2005. HeLa cells transfected with pcDNAcHA-Arf1 were lysed in 20 mM Tris-HCl (pH7.4), 100 mM NaCl, 4 mM MgCl_2_, 0.2% (v/v) T×100, 0.5 mM DTT, and Complete EDTA-free protease inhibitor cocktail (Roche), and lysates were centrifuged (16,000 g, 10 min) to isolate detergent-soluble fraction. Purified GST-GGA3-GAT^1-313^ (50 μg) was incubated with the detergent-soluble fraction (1 hr, 4°C) then HA-tagged Arf1 immunoprecipitated with HA-agarose (25 μl, 4°C, 2 hr). HA-agarose beads were washed and bound proteins were eluted with SDS-UREA sample buffer, then samples were immunoblotted using HA and GST antibodies.

See also Supplemental Experimental Procedures.

## Supplemental Information

Document S1. Supplemental Experimental Procedures, Five Figures, and Two Tables
