# Supplementary material for: Salmonella Virulence Effector SopE and Host GEF ARNO Cooperate to Recruit and Activate WAVE to Trigger Bacterial Invasion
Source: Cell Host Microbe. 2012 Feb 16;11(2):129–39. doi: 10.1016/j.chom.2012.01.006 (PMC3314957; doi:10.1016/j.chom.2012.01.006)
Supplement: Document S1. Supplemental Experimental Procedures, Five Figures, and Two Tables [file mmc1.pdf]

Cell Host & Microbe, Volume *11*

Supplemental Information

Salmonella Virulence Effector SopE and Host GEF ARNO Cooperate to Recruit and Activate WAVE to Trigger Bacterial Invasion

Daniel Humphreys, Anthony Davidson, Peter J. Hume, and Vassilis Koronakis

Supplemental Experimental Procedures

Plasmids and Recombinant Proteins

The following plasmids were generated by Invitrogen Gateway methodology: pcDNA3.1-SopE$^{78\text{-}241}$-FLAG, pcDNA3-mRFP-Arf1, pcDNAcHA-Arf1, peCFP-C1-HSPC300, peCFP-C1-GGA3-GAT$^{1\text{-}313}$, pGEX2T-GGA3-GAT$^{1\text{-}313}$ and peCFP-C1-ARNO. Point mutations were introduced into *arno* by site-directed mutagenesis. The vector pGST:SopE$^{78\text{-}241}$ has been described (Cain et al., 2004). The following were kindly provided to us; pRSETc-ARNO-2G (Prof Shamshad Cockcroft), pET-arf1 and pBB131 encoding the Arf family N-myristoyltransferase (Prof Martin Spiess). GST- and His-tagged proteins were expressed in *E.coli* Rosetta (Novagen) at 16°C before affinity purification (Cain et al., 2004).

Antibodies

Antibodies were purchased from Abcam (Cdc42, ab64533: Rac1, ab33186; Arf1, ab58578; Arf6, ab81650; HSPC300, ab87449; ARNO, ab56510; Big1, ab72061; Big2, ab75001), Sigma (Abi1, A5106; Cyfip, P0092; Nap1, N3788; FLAG, F1804; HA agarose, A2095), Santa Cruz (HSP90, sc7947), GE Healthcare (GST, 27-4577-01) Covance (HA, MMS-101R) or were raised against recombinant peptides in rabbits by Diagnostics Scotland (WAVE2, amino acids 180-241).

Mammalian Cell Culture and Transfections

Mammalian HeLa cells (ATCC-CCL-2) were routinely cultured in complete growth media consisting of Dulbecco's modified Eagle's medium (DMEM) supplemented with 10% (v/v) fetal bovine serum (FBS), 2mM L-glutamine, 200μg/ml^{-1} streptomycin and 100Uml^{-1} penicillin (37°C, 5% CO_2). Transient transfection of HeLa cells by microporation was performed using the Neon Transfection System according to manufacturers instructions (Invitrogen). For RNA-interference small-interfering RNA (siRNA) from Qiagen against Arf1 (gene accession number NM_001024228, Hs_Arf1_1 Seq: ACGTGGAAACCGTGGAGTACA, Hs_Arf1_11 Seq: AGGGAAGACCACGATCCTCTA, Hs_Arf1_10 Seq: CACGATCCTCTACAAGCTTAA), Rac1 (gene accession number NM_006908, Hs_RAC1_5 Seq: ATGCATTTCCTGGAGAATATA), ARNO (gene accession number

NM_004228, Hs_PSCD2_2 Seq: CTGGTTTATCCTCACAGACAA, Hs_PSCD2_3 Seq: CACGCTGTTGGTAATCTTATT, Hs_PSCD2_6 Seq: ATGGAGGACGGCGTCTATGAA, Hs_PSCD2_7 Seq: CAGTAAGACCTTGCAACGGAA), GBF1 (gene accession number NM_004193, Hs_GBF1_3 Seq: CAGGAGCATGTACATATGGAA, Hs_GBF1_4 Seq: AAGGAAGACCTTACTGATCTA), Big1 (gene accession number NM_006421, Hs_ARFGEF1_5 Seq: ACTGTTGATCATATATCCCA, Hs_ARFGEF1_6 Seq: CGCGTTGATACTCAAGACCAA) Big2 (gene accession number NM_006420, Hs_ARFGEF2_6 Seq: CGGCGGCTGGCTGTACAACTTA, Hs_ARFGEF2_7 Seq: GTGGCGCTCGATGAAATTAAA) or Allstars negative control siRNA (Qiagen), were transfected into HeLa cells with Oligofectamine™ transfection reagent (Invitrogen) according to manufacturer's instructions. Transfection mixture was replaced after 24h with complete growth medium and cells cultured 72 hours in total. RNAi efficiency was determined by EXPRESS One-Step SYBR® GreeenER qRT-PCR according to manufacturers instructions (Invitrogen).

Protein Identification by Mass Spectrometry

Proteins were separated on 9% Bis-Tris gels in MOPS pH7.6 running buffer. Excised protein bands from colloidal Coomassie Blue G250-stained gels were reduced, carboxymethylated, digested using trypsin and identified by electrospray ionization liquid chromatography mass spectrometry (Mass spectrometry service, Cambridge Centre for Proteomics, University of Cambridge). The MS fragmentation data was used to search the National Center for Biotechnology Information database (NCBI) using the MASCOT search engine (http://www.matrixscience.com). Probability-based MASCOT scores were used to evaluate identifications. Only matches with $p < 0.05$ for random occurrence were considered significant.

Table S1. Proteins Recruited from Extract to Membranes by SopE

The table lists identified proteins recruited by SopE-decorated PL beads from extract with added GTPγS. The data corresponds to the gel bands (indicated by orange circles) and mass spectrometry shown in Figure 1A. Probability-based MASCOT scores were used to evaluate identifications. Only matches with $p < 0.05$ for random occurrence were considered significant.

**Gel Band Figure 1A**	**Protein Name**	**Peptide Coverage (%)**	**Mascot Score**	**MW (kD)**
Cyfip	cytoplasmic FMR1 interacting protein 1/2	53.3/ 44.4	3044/2798	~147
Nap1	NCK-associated protein 1	52.6	2579	~129
WAVE	WASP family Veroprolin homologue member 1/2/3	20.8/7.6/32.4	452/197/559	~62/ ~54/51
Abi	abl-interactor 2	25.8	549	~52
N-WASP	Neuronal-wiskott-Aldrich syndrome protein	14.2	297	~53
Cdc42	cell division cycle 42	43.5	386	~22
Rac	ras-related C3 botulinum toxin substrate 1/2/3	67.4/36.5/46.9	347/314/436	~22
Arfs	ADP-ribosylation factor 1/2/3/4/5/6	80.6/59.7/ 80.1/45/60/30.3	968/831/971/ 427/763/265	~20
actin	actin	42.1	561	~40

Figure S1

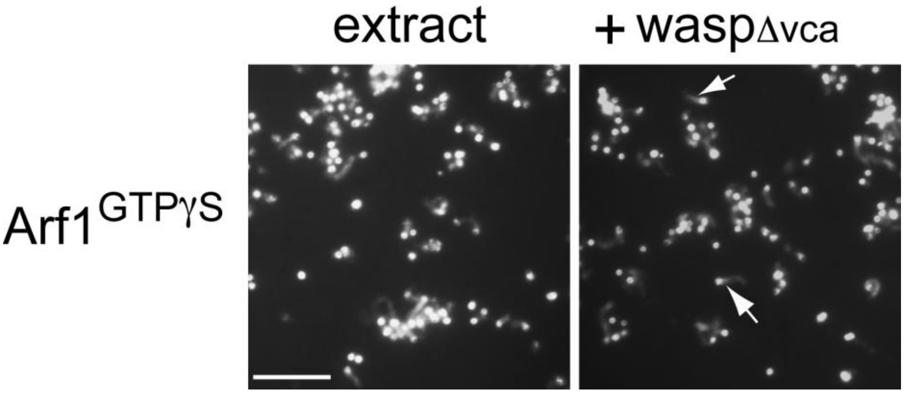

Figure S1. Arf1 Activation of WRC

Fluorescence microscopy of rhodamine-actin assembly on the PL beads with anchored active myristolyated Arf1$^{GTP\gamma S}$ in extract with or without waspΔvca. Scale bar 15μm. Control experiment for Figure 2B.

Figure S2

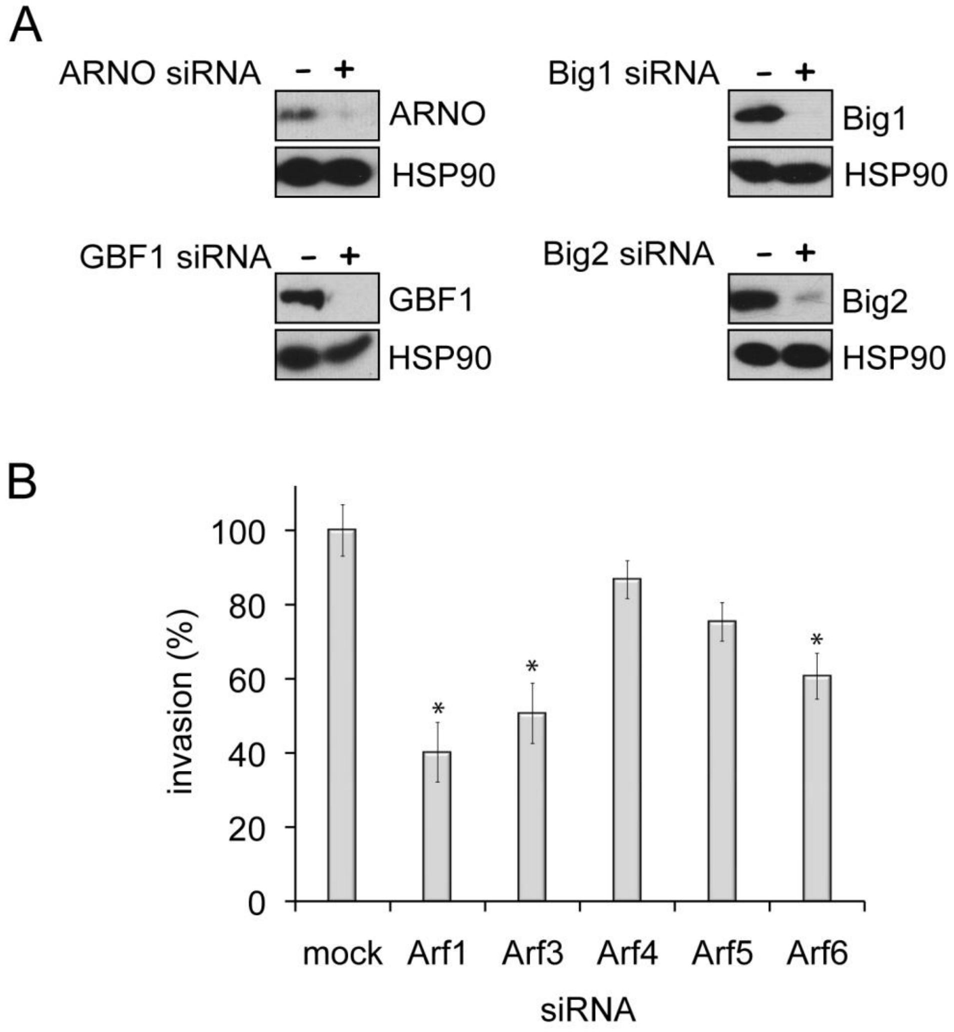

Figure S2. Arf GEF Depletion and Arf GTPase Promotion of *Salmonella* Invasion

(A) Arf GEF depletion by siRNA transfection. HeLa cells were transfected with either ARNO, GBF1, Big1 or Big2 siRNA 72h before analysis by SDS-PAGE and immunoblotting with indicated antibodies to Arf GEFs and HSP90 as control (right).

(B) Influence of Arf GTPase depletion on *Salmonella* invasion. HeLa cells were transfected with either mock or indicated Arf siRNA 72h before infection with wt *Salmonella* (15 min). To quantify *Salmonella* invasion, washed infected cells were incubated with gentamicin for 2h to kill extracellular bacteria and invasion quantified by colony counts. Asterisks indicate a significant difference from mock ($p < 0.05$, ANOVA; n=3). Error bars represent +/- SEM. Knockdowns were quantified by qRT-PCR, data not shown. Experiments correspond to Figure 4.

FigureS3

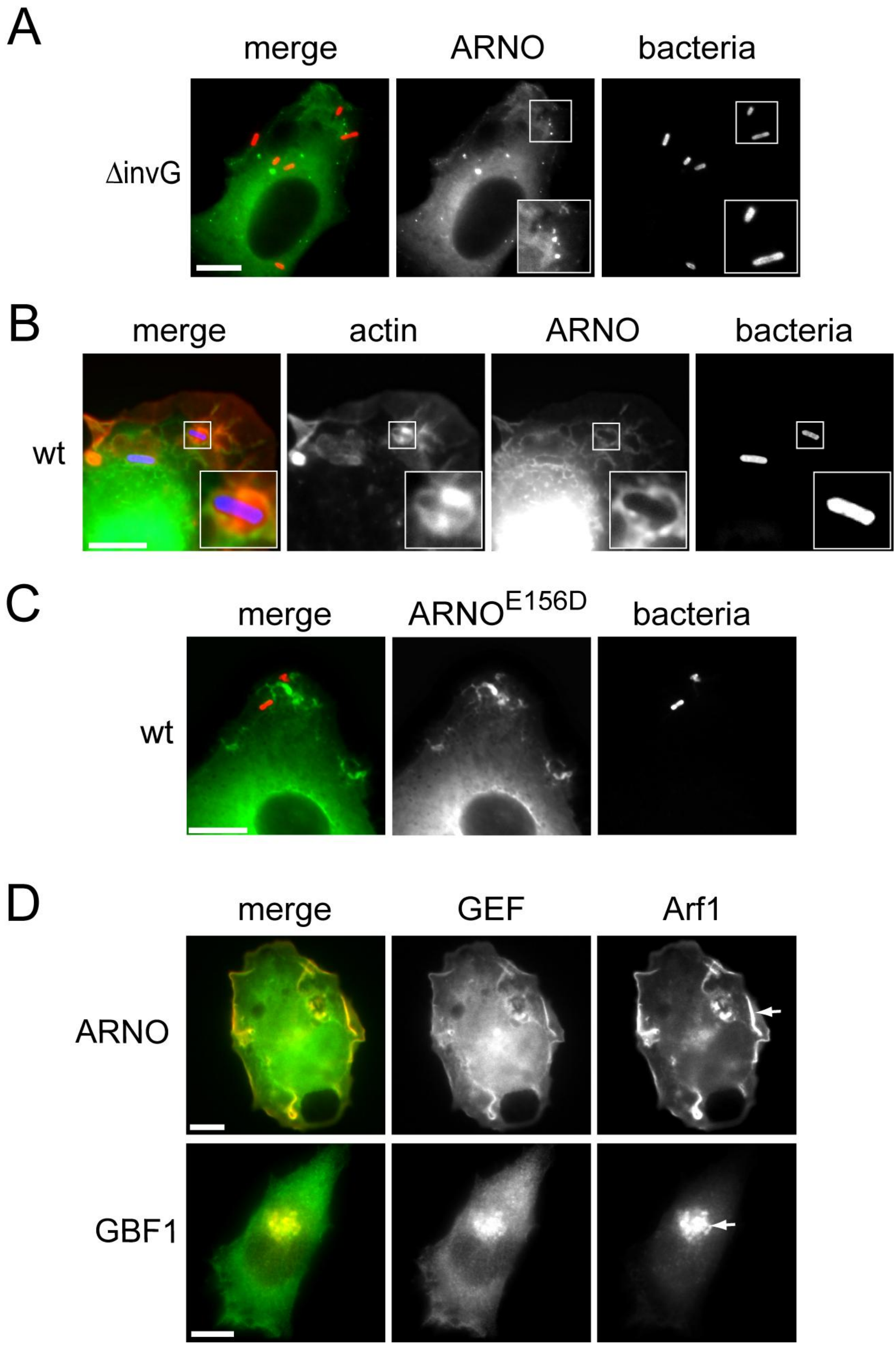
A
merge
ARNO
bacteria
ΔinvG
B
merge
actin
ARNO
bacteria
wt
C
merge
ARNO^{E156D}
bacteria
wt
D
merge
GEF
Arf1
ARNO
GBF1

Figure S3. ARNO Localization and Arf1 Recruitment to the Plasma Membrane

(A) Localization of ARNO during infection with *ΔinvG Salmonella*. Like Figure 5a, HeLa cells expressing ^{CFP}ARNO (ARNO) were infected for 15 min with fluorescently-labelled *ΔinvG Salmonella* (bacteria). Scale bar 8μm. Insets show magnified area.

(B) Localization of ARNO and actin during *Salmonella* invasion. HeLa cells expressing ^{CFP}ARNO (ARNO) were infected for 15 min with fluorescently-labelled wt *Salmonella* (bacteria) as indicated (left) then stained with TexasRed-phalloidin to label actin. Insets magnify ARNO and actin co-localization at a pathogen containing macropinosome.

(C) Macropinosome formation by catalytically inactive ARNO during *Salmonella* invasion. HeLa cells expressing ^{CFP}ARNO^{E156D} (ARNO^{E156D}) were infected for 15 min with fluorescently-labelled wt *Salmonella* (bacteria) as indicated (left).

(D) Influence of Arf GEF expression on Arf1 localization. HeLa cells expressing Arf1^{RFP} together with ^{CFP}ARNO or ^{CFP}GBF1. Arrows indicate enriched Arf1 localization. Scale bars 8μm. Experiments correspond to Figure 5.

Table S2. Proteins Recruited from Extract to Membranes by ARNO

The table lists identified proteins recruited by ARNO-decorated PL beads from extract with added GTPγS. The data corresponds to the gel bands (indicated by orange circles) and mass spectrometry shown in Figure 6A. Probability-based MASCOT scores were used to evaluate identifications. Only matches with $p < 0.05$ for random occurrence were considered significant.

Gel Band Figure 6A	Protein Name	Peptide Coverage (%)	Mascot Score	MW (kD)
Cyfip	cytoplasmic FMR1 interacting protein 1/2	47/ 39	3509/2637	~147
Nap1	NCK-associated protein 1	43	2573	~129
WAVE	WASP family Veroprolin homologue member 1/2/3	28/6/30	870/136/831	~55
Abi	abl-interactor 1/2	24/27	358/534	~52
N-WASP	Neuronal-wiskott-Aldrich syndrome protein	15	303	~53
Cdc42	cell division cycle 42	41	223	~22
Rac	ras-related C3 botulinum toxin substrate 3	22	166	~22
Arfs	ADP-ribosylation factor 1/2/3/4/5/6	59/75/ 79/58/58/19	824/952/785/ 630/608/180	~20
actin	actin	22.1	254	~40

Figure S4

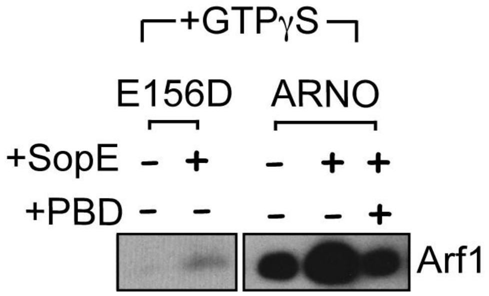

Figure S4. SopE Promotion of Arf1 Recruitment

Arf1 recruitment to phospholipid bilayers by SopE in the presence of ARNO. PL beads decorated with either catalytically-inactive ARNO (E156D) or wild-type ARNO with or without SopE were isolated from brain extract containing GTPγS in the presence or absence of the Rho GTPase inhibitor PBD. Recruited proteins were extracted before analysis by SDS-PAGE and immunoblotting with Arf1 antibodies (right). Control experiment for Figures 6 and 7.

The left panel demonstrates that catalytically-inactive ARNO (E156D) cannot recruit Arf1 but Arf1 recruitment is observed when SopE and E156D are combined. The right panel re-confirms that Arf1 recruitment is increased when SopE and ARNO are together (also shown in Figure 7A). The increase is blocked by PBD. These results indicate that SopE promotes Arf1 recruitment via activation of Rho GTPases and not via enhanced ARNO GEF activity in the presence of SopE or WRC.

Figure S5

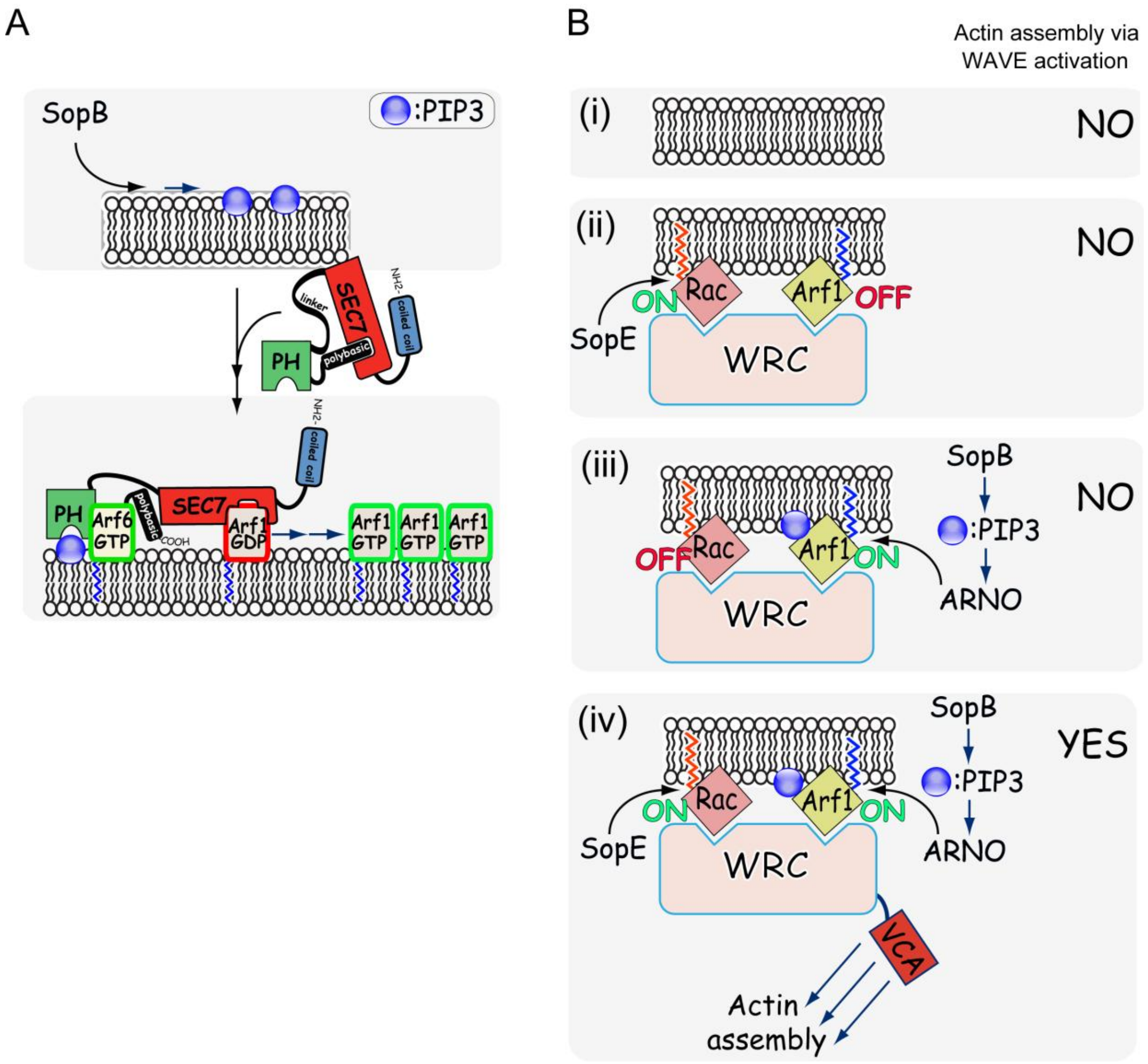

Figure S5. Recruitment of ARNO, which Cooperates with SopE to Activate WRC

(A) ARNO recruitment by *Salmonella*. ARNO adopts an autoinhibited conformation (top) where the polybasic motif and linker mask catalytic Sec7 domain (Donaldson and Jackson, 2011). ARNO recruitment to the plasma membrane depends upon its PH domain that binds the phosphoinositide PIP3 and Arf6 (Cohen et al., 2007; Klarlund et al., 2000; Macia et al., 2000). *Salmonella* SopB is known to trigger generation of PIP3 at invasion ruffles (Mallo et al., 2008), and we show that SopB-induced PIP3 production (top) and ARNO interactions with PIP3 and Arf6 are crucial for *Salmonella* to recruit ARNO and facilitate Arf1 activation (bottom). Corresponds to the data in Figure 5B and 5C.

(B) Cooperation of SopE and ARNO in recruitment and activation of WRC.

(i) Neither recruitment nor activation of WRC at the membrane is observed in the absence of active Arf1and Rac1, which blocks actin assembly.

(ii) SopE activation of Rho GTPases such as Rac1 (ON) triggers recruitment but not activation of WRC, as Arf1 remains inactive (OFF), so no WRC-dependent actin assembly occurs.

(iii) SopB promotes recruitment of ARNO that activates Arf1 (ON) thereby triggering recruitment but not activation of WRC, as Rac1 remains inactive (OFF), which prohibits robust WRC-dependent actin assembly.

(iv) SopB promotes recruitment of ARNO which cooperates with SopE to activate (ON) Arf1 and Rac1, respectively, thereby triggering recruitment and activation of WRC for initiation of actin assembly via the WAVE VCA domain. Corresponds to the data in Figure 7.
